# Second International Symposium on Recent Advances in Environmental Health Research

**DOI:** 10.3390/ijerph2006030000

**Published:** 2006-03-31

**Authors:** Abdul K. Mohamed, Paul B. Tchounwou

**Affiliations:** 1 Dean and Director, National Institutes of Health RCMI-Center for Environmental Health.; 2 Chair and Deputy Director, National Institutes of Health RCMI-Center for Environmental Health.

The *Second International Symposium on Recent Advances in Environmental Health Research* was organized by Jackson State University (JSU) from September 18 – 21, 2005 at the Regency Hotel in Jackson, Mississippi. The symposium was built upon the overwhelming success of the 2004 conference that was also organized by JSU and co-sponsored by the National Institutes of Health (NIH) RCMI-Center for Environmental Health, the U.S. Department of Education Title III Graduate Education Program, the U.S. Environmental Protection Agency, and the JSU office of Research. This international meeting commemorated 20 years of the NIH-NCRR Research Centers in Minority Institutions (RCMI) Program, a congressionally mandated program initiated by the NIH in 1985 with the mission to expand the national capability for research in the health sciences. Over the years, RCMI institutions have played a significant role in addressing important biomedical research questions that are critical in managing the health needs of the nation in a cost-effective manner. At JSU, the RCMI program has served as a catalyst for excellence in biomedical research and education. Research conducted through the RCMI Program is providing a strong scientific basis for understanding the pathogenesis and for discovering innovative therapies for human diseases such as cancer and AIDS. Through the RCMI Program, doctoral students trained at JSU have gone on to obtain competitive post-doctoral positions at prestigious institutions such as Harvard Medical School, John Hopkins Medical Center, Purdue University Medical Center, and the NIH. As echoed by President Ronald A. Mason, Jr., *“*…*JSU is proud to be integral part of the very successful and productive RCMI Program. This symposium highlighted the excellence in biomedical, clinical, and behavioral research conducted by RCMI institutions*…*”*

In an attempt to contribute global solutions to these environmental challenges, scientists around the world, have been more and more involved in bioenvironmental research, studying the toxic mechanisms of action of various environmental agents, developing new approaches for detecting or remedying environmental damage, identifying and characterizing genes involved in the manifestation of environmentally-related diseases, and providing the public and policy makers with scientific tools that are critical for environmental health decision-making.

Building on the foundation of the first symposium, the *Second International Symposium on Recent Advances in Environmental Health Research* served as a platform for environmental and biomedical scientists-biologists, chemists, toxicologists, public health scientists, engineers, and policy makers interested in bringing about substantial contributions to addressing global environmental, and sustainable development issues, to communicate the latest advances in scientific research and new developments on critical environmental and human health topics including the following:

1. ***New Frontiers in Environmental Health Research***: The causes of most human diseases have been attributed to the complex interactions between genetic factors and environmental exposures. Hence, control and prevention measures highly rely on the understanding of the cause and effect relationships between these factors and disease development. In recent years, new areas of research such as toxicogenomics, proteomics, and functional genomics have emerged, with the aim of understanding molecular mechanisms of health and disease. Also, the recent advances in the molecular biology of the cell cycle regulation have given new life to our understanding of cancer in particular, and the idea that defects of regulation in cancer cells may partially explain successes that have been achieved in cancer chemotherapy. Specific areas of symposium research presentations included gene expression studies, proteomics, gene-environment interactions, functional genomics, biomarkers of effect, sensitivity and effect, signal transduction and gene activation; and molecular targets of disease chemotherapy.
2. ***Environmental Toxicology and Health Risk Assessment***: Growing public awareness of the potential risk to humans from toxic chemicals in the environment has generated demand for new and improved methods for toxicity assessment and rational means for estimating health risk. Many environmental agents such as metal ions, polycyclic aromatic hydrocarbons, pesticides/herbicides, UV-light, food additives, and viruses are known to induce various types of illnesses including cancer in humans. Several symposium presentations dealt with research elucidating the cellular and molecular mechanisms by which these environmental agents induce toxicity, mutagenesis, and carcinogenesis, as well as research on hazard assessment of exposure to physical, chemical and biological agents; dose-response evaluation and model development; exposure assessment analysis; and health risk characterization; and management.
3. ***Emerging Topics in Computational Biology, and Environmental Modeling***: Using of computational methods and procedures to investigate environmental and biological phenomena has made remarkable progresses. This field includes analysis of human genome data, prediction of DNA and protein structure and function, design of biomaterials and therapeutic agents, studies into small molecule-biomacromolecule interactions, and other related computational method development. Therefore, several symposium presentations dealt with the computational analysis of the physical and chemical properties of several environmental compounds, as well as on quantitative structure activity relationship (QSAR) studies for developing predictive toxicology models associated with exposure to these compounds.
4. ***Health Disparities and Environmental Security***: In recent years health disparities and biological and chemical terrorism have emerged as major issues in public safety and homeland security. With recent advances in laboratory technologies, it is often possible to measure specific genetic variations as risk factors for specific types of disease. Equally important is the evaluation of the role of modifier factors such as environmental exposures or other genes that may exacerbate the genetic risk leading to differences in disease susceptibility among individuals. Since the events of September 11, 2001 regarding the attacks on the World Trade Center and the Pentagon, and the subsequent anthrax attacks on several people, our collective thinking with regard to our vulnerability to terrorism has completely changed. The specific areas of research presentations included the following: health disparities and cancer; health disparities and heart disease; health disparities and infectious diseases; and bioterrorism/chemical terrorism.
5. ***Medical Geology and Human Health***: Recent concerns over health-related issues arising from exposure to environmental substances have raised substantial interest in a new field termed “medical geology”. In fact, naturally-occurring toxic metals such as arsenic, cadmium, lead, and mercury are now known to cause serious public health problems in several areas of the world. Likewise, the geographical distributions of several infectious diseases such as malaria, meningitis, and schistosomiasis, have been linked to intrinsic climatic and environmental factors. Research on this topic dealt with disease ecology, toxicology, pathology and/or epidemiology with regard to the emerging subject of medical geology.
6. ***Natural Resources Damage Assessment and Management***: Several environmental influences including natural and anthropogenic factors have been linked to ecosystem vulnerability. Monitoring and assessment data are therefore needed for science-based decision-making with regard to environmental management. Papers for presentation on this topic included those related to: a) conceptual modeling for ecological risk assessment, b) assessment of the physical, chemical, and biological characteristics of specific ecosystems, c) applications of GIS and remote sensing technology to environmental assessment and management, and d) bioindicators for environmental management.

The symposium attracted 260 participants from 21 countries representing all five continents, and more than 150 scientific presentations across the disciplines of environmental health and biomedical sciences. As stated above, the scientific program was composed of six plenary sessions where oral/platform presentations were given by more than 40 invited speakers. In addition, there were two poster sessions – one for faculty and professional scientists, and one for students – with more than 100 abstracts. The submitted full length manuscripts were peer-reviewed, and selected for publication by experts in their respective fields. The accepted papers are being published in three volumes as special issues of the International Journal of Environmental Research and Public Health.

We wish to extend special thanks to Dr. Sheila McClure, Director of the NIH-NCRR-RCMI Program, Bethesda, Maryland, for serving as Banquet Speaker, and Dr. James Townsel, Director of the RCMI Center for Molecular Neurosciences at Meharry Medical College, Nashville, Tennessee, for serving as Distinguished Speaker for the Second Honorary Biomedical and Health Information Lecture Series” at the symposium. He made a distinguished lecture on the Commitment to Eliminate Health Disparities and the Need to Achieve Diversity within the Biomedical Workforce. Thanks are also extended to all our conference presenters, session chairs, and keynote speakers, and especially Dr. John McLachlan, Distinguished Professor of Environmental Studies at Tulane University, and Director of the Center for Bioenvironmental Research at Tulane/Xavier Universities, for giving the inaugural presentation on *Environmental Endocrine Disrupting Chemicals and Epigenetic Gene Imprinting;* focusing on the new mechanisms to understand the environmental component of human disease. Many thanks to Mrs. Zelma Leflore and her students for providing the technical assistance with the technology needed for platform presentations, emails, and other services.

Special thanks are extended Dr. Ronald Mason, Jr. (President), Dr. Felix Okojie (Vice President for Research Development, and Federal Relations), Dr. Velvelyn Foster (Interim Provost and Vice-President for Academic Affairs), and Dr. Mary Myles (Director of Title III Program) for their administrative support. We would like to acknowledge the authors for their involvement and cooperation, and for their outstanding contributions to advancing science and sound decision-making in the critical area of environmental health sciences. Special thanks are also extended to all the peer-reviewers who took time off their busy schedules to carefully and critically review each of the manuscripts.

On behalf of the entire organizing committee, the greatest acknowledgments go to our major symposium sponsors including the U.S. Department of Education Title III-Strengthening the Environmental Science Ph.D. Program at JSU, National Institutes of Health RCMI-Center for Environmental Health, and JSU Office of Research Development and Federal Relations.

